# Treatment-related biomarkers in pulmonary hypertension patients on oral therapies

**DOI:** 10.1186/s12931-020-01566-y

**Published:** 2020-11-19

**Authors:** Aparna C. Swaminathan, Hongmei Zhu, Victor Tapson, Yuliya Lokhnygina, Abby Poms, Zach Kelleher, Elijah Gaspard, Karla Kennedy, Brian E. Fee, Terry Fortin, S. Nicholas Mason, Kishan Parikh, Tim J. McMahon

**Affiliations:** 1 Department of Medicine, Duke University Medical Center, DUMC 2650, 203 Research Drive, Durham, NC 27710 USA; 2 Department of Medicine, Cedars-Sinai Medical Center, Los Angeles, CA 90048 USA; 3 Department of Biostatistics and Bioinformatics, Duke University Medical Center, Durham, NC 27710 USA; 4 Departments of Medicine and Research and Development, Durham VA Healthcare System, Durham, NC 27705 USA

**Keywords:** Pulmonary hypertension, Biomarkers, Nitric oxide, Endothelin-1

## Abstract

**Background:**

Multiple classes of oral therapy are available for the treatment of pulmonary arterial hypertension (PAH), but there is little to guide clinicians in choosing a specific regimen or therapeutic class. We aimed to investigate whether treatment-relevant blood biomarkers can predict therapy response in prevalent PAH patients.

**Methods:**

This prospective cohort study longitudinally assessed biomarkers along the endothelin-1 (ET-1) and nitric oxide (cGMP, ADMA, SDMA, nitrite, and *S*-nitrosohemoglobin) pathways along with the cGMP/NT-proBNP ratio over 12 months in patients with WHO Group 1 PAH on oral PAH-specific therapies. The relationship between biomarkers and 6MWD at the same and future visits was examined using mixed linear regression models adjusted for age. As cGMP can be elevated when NT-proBNP is elevated, we also tested the relationship between 6MWD and the cGMP/NT-pro BNP ratio. Patients with PAH with concomitant heart or lung disease or chronic thromboembolic pulmonary hypertension (CTEPH) were included in a sensitivity analysis.

**Results:**

The study cohort included 58 patients with PAH treated with either an endothelin receptor antagonist (27.6%), phosphodiesterase-5 inhibitor (25.9%) or a combination of the two (43.1%). Among biomarkers along the current therapeutic pathways, ET-1 and the cGMP/NT-proBNP ratio associated with same visit 6MWD (p = 0.02 and p = 0.03 respectively), and ET-1 predicted future 6MWD (p = 0.02). ET-1 (p = 0.01) and cGMP/NT-proBNP ratio (p = 0.04) also predicted future 6MWD in the larger cohort (n = 108) of PAH patients with concomitant left heart disease (n = 17), lung disease (n = 20), or CTEPH (n = 13). Finally, in the larger cohort, SDMA associated with 6MWD at the same visit (p = 0.01) in all subgroups and ADMA associated with 6MWD in PAH patients with concomitant lung disease (p = 0.03) and PAH patients on ERA therapy (p = 0.01).

**Conclusions:**

ET-1, cGMP/NTproBNP ratio, and dimethylarginines ADMA and SDMA are mediators along pathways targeted by oral PAH therapies that associate with or predict 6MWD.

## Background

Pulmonary arterial hypertension (PAH) carries significant morbidity and mortality and is characterized by progressive elevation of pulmonary arterial pressures and secondary right heart failure [1]. The pathogenesis of PAH is complex and involves metabolic and cellular dysfunction, inflammation, fibrosis and dysregulation of vasoactive and vasoproliferative mediator axes [2]. Current therapies for PAH generally target abnormalities in one of three intracellular pathways with signaling dysfunction: the prostacyclin, nitric oxide (NO), or endothelin (ET) pathway. Over the past 20 years, 14 new therapies have been approved by the US Food and Drug Administration to treat PAH, but no optimal regimen or therapeutic class has been identified. While improved outcomes have been seen with initial combination therapy in PAH [3, 4], this approach markedly increases cost of treatment and the burden of therapy to the patient in terms of compliance and side effects. In addition, there may be subsets of PAH patients in whom initial monotherapy is sufficient [5]. There is a pressing need to better predict treatment response in order to personalize and otherwise guide selection among the existing therapies [6, 7].

Objective and practical biomarkers may help predict response to a therapeutic intervention in an individual patient. While a variety of biomarkers have been explored in PAH, brain natriuretic peptide (BNP) and the N-terminal fragment (NT) of pro-BNP (NT-proBNP) remain the only biomarkers recommended by current guidelines for risk stratification and assessing response to treatment [8, 9]. Given that the currently available oral therapies act on targets in either the NO/soluble guanylate cyclase (sGC)/cyclic guanosine monophosphate (cGMP) pathway, ET pathway, or prostanoid/cyclic adenosine monophosphate pathways, it is plausible that biomarkers in these pathways will predict optimal therapy for maximal treatment response. However, interactions among circulating mediators that can serve as treatment-related biomarkers are complex. For example, BNP elevates cGMP, and ET-1 can stimulate NO synthesis, which can elevate levels of NO derivatives nitrite (NO_2_^−^) and S-nitrosohemoglobin (SNO-Hb) [10–13]. This suggests that examining these biomarkers in concert may have value, and that biomarker utility could be unmasked when one biomarker is normalized to another, covarying biomarker. Although some prior work has examined the utility of individual biomarkers with potential relevance to therapy choice [13], no study has investigated these biomarkers in parallel.

We aimed to prospectively investigate the clinical utility of treatment-related biomarkers that reflect or interact with the biological pathways targeted by existing therapies in patients with prevalent pulmonary vascular disease taking targeted oral PAH therapies. Specifically, we assayed the following biomarkers along the NO or ET-1 pathways: ET-1, cGMP, asymmetric dimethylarginine (ADMA), symmetric dimethylarginine (SDMA), NO_2_^−^, and SNO-Hb. We also tested the relationship between the cGMP: NT-proBNP ratio, a pre-specified biomarker variable, and the study outcomes as a test case of whether accounting for this relationship might enhance a biomarker finding. In addition, we studied NT-proBNP given its role as described in current guidelines, and red blood cell distribution width (RDW), which is commonly reported along with routine blood count tests and appears to have biomarker utility in PAH [14]. These two biomarkers served as study benchmarks or internal controls. We hypothesized that (1) certain biomarkers associated with treatment classes (e.g., cGMP, ET-1) would predict the current or next-visit six minute walk distance (6MWD); and (2) the relationship between each biomarker and 6MWD would vary depending on the class of PH-targeted therapy and/or PH etiology.

## Study design and methods

### Study cohort

This study included patients with PAH seen in Duke University Medical Center’s Pulmonary Vascular Disease Center already on targeted oral PAH therapy. Study entry was not dependent on or accompanied by the start of any new therapy or change in therapy. PAH was defined by the following hemodynamic criteria: mean pulmonary artery pressure (mPAP) ≥ 25 mm Hg, pulmonary capillary wedge pressure (PCWP) ≤ 15 mm Hg, and pulmonary vascular resistance (PVR) ≥ 3 Woods units. Additional sensitivity analyses also included patients with PAH who had concomitant left heart disease or lung disease as well as patients with chronic thromboembolic pulmonary hypertension (CTEPH) on oral PAH therapy. PAH with concomitant left heart disease was differentiated from World Health Organization (WHO) Group 2 PH by the treating clinician based on elevated transpulmonary and diastolic pulmonary gradients [6] and specifically defined by the following criteria: mPAP ≥ 25 mm Hg, PCWP ≥ 15 mm Hg, transpulmonary gradient > 12 mmHg, diastolic pulmonary gradient > 5 mmHg, *and* PVR ≥ 3 Wood units. PAH with concomitant lung disease was differentiated from WHO Group 3 PH by the treating clinician based on the following hemodynamic and clinical criteria: (1) mPAP ≥ 40 mm Hg along with either a low cardiac index (< 2 L/min/m^2^) or RV dysfunction on echocardiogram; (2) minimal or only modest airway or parenchymal abnormalities on imaging; and (3) mild or moderate impairment on physiologic testing.

All care was provided according to standard practice, with no requirements regarding therapeutic changes or diagnostic procedures dictated by the protocol. Visit intervals were calculated and categorized based on their proximity to 3, 6, 9, 12, or 18 months from study enrollment. This study was approved through the Duke University institutional review board (Duke IRB Protocol Pro00046651).

Patients were followed for at least one year and at each routine visit, we assayed blood for the following biomarkers: ET-1, cGMP, ADMA, SDMA, SNO-Hb, NO_2_^−^, NT-proBNP, cGMP:NT-proBNP ratio, and RDW. No other biomarker ratios were examined. We also identified PAH-related clinical events, defined as death, lung transplant, PAH-related hospitalization, initiation of subcutaneous, intravenous, or inhaled prostacyclin, or addition of oral therapy due to clinical worsening, that occurred within six months of a biomarker value.

### Study outcomes

The outcomes of this study included 6MWD at the same visit and 6MWD at the subsequent visit.

### Biomarker assays

ADMA, SDMA, ET-1 and cGMP were assayed using ELISA kits. Plasma and RBCs were separated centrifugally. SNO-Hb was assayed using the validated mercury-coupled photolysis-chemiluminescence technique as we previously described [15]. Blood was collected in specific EDTA-containing Vacutainer tubes demonstrated to contain the lowest values of contaminant nitrite among commercial tubes. Samples were protected from light and transported and processed within 120 min (range, 26–114 min to assay initiation or freeze; mean ± SD: 61.7 ± 18.2 min). RBC SNO-Hb was assayed immediately in > 90% of instances, and other biomarkers were deep-frozen for later batch analysis. Nitrite (NO_2_^−^) was measured using chemical reduction (triiodide) coupled with chemiluminescence (Sievers NO Analyzer 280, Boulder, CO).

### Statistical analysis

The univariable associations between biomarker values and same-visit 6MWD were assessed using mixed linear regression models in order to account for correlations between multiple observations from each patient. Values of ET-1, NT-proBNP, RDW, cGMP, SNO-Hb, SDMA, cGMP/NTproBNP ratio, and NO_2_^−^ were log-transformed to improve the distributional assumptions. Linearity of the association with 6MWD was checked for each biomarker. If the linearity assumption did not hold, the nonlinear relationship was approximated using a piecewise-linear spline. All models were adjusted for age. Interaction terms were added to the models to allow the association between 6MWD and each biomarker to differ between the following groups: PAH patients on endothelin receptor antagonist (ERA), PAH patients on phosphodiesterase 5 inhibitor (PDE5i), and PAH patients on combination of ERA and PDE5i.

Mixed linear regression models were used to determine the relationship between each biomarker and next-visit 6MWD and adjusted for age. Missing biomarker values were imputed in this analysis using the last value carried forward.

A sensitivity analysis was performed to evaluate the relationship between the above biomarkers and 6MWD in a larger cohort of patients that included not only the original group of PAH patients, but also PAH patients with concomitant left heart disease or lung disease being treated with oral PAH directed therapy, along with CTEPH patients on medical therapy. Similar to the primary analysis, mixed linear regression models adjusted for age were used to evaluate the relationship with each biomarker and 6MWD at the same visit, or 6MWD at the subsequent visit. In the sensitivity analysis, additional interaction terms were added to allow the association between 6MWD and each biomarker to differ between the following groups: PAH patients on ERA, PAH patients on PDE5i, PAH patients on combination of ERA and PDE5i, PAH patients with concomitant heart disease, PAH patients with concomitant lung disease, and patients with CTEPH.

A multivariable model was created in this larger cohort, correlating biomarkers with same visit 6MWD using forward selection, with p ≤ 0.05 as criterion to enter the model.

## Results

### Study cohort

Demographics and baseline characteristics of the 58 PAH patients enrolled in the study are detailed in Table 1. In brief, the cohort was predominantly female (89.7%) with a median age of 61. The median pulmonary vascular resistance was 5.1 woods units (IQR 3.6, 6.3) and median 6MWD 427.9 m (IQR 362.1, 487.4). With regards to treatment, 27.6% of patients were treated with an ERA, 25.9% with a PDE5i, and 43.1% with a combination of an ERA and PDE5i. In addition, 2 patients (3.4%) were on monotherapy with sGC and 1 patient (1.7%) was on combination ERA and sGC therapy. At study baseline, ET-1 levels were highest in patients on a combination of ERA and PDE-5i, and lowest in patients on PDE-5i monotherapy. Nitrite levels were also lowest among patients on PDE-5i monotherapy, and highest in patients on ERA. In contrast both cGMP and SDMA levels were lowest in patients on ERAs, and highest in patients on PDE-5i.

**Table 1:** Baseline patient characteristics and biomarker values

	Overall N = 58	On therapy with ERA N = 16	On therapy with PDE5i or sGC^a^ N = 17	On therapy with ERA + PDE5i^b^ N = 25
Age (years)	61.1 (46.1, 68.5)	61.6 (51.0, 68.1)	69.1 (61.1, 76.4)	51.2 (37.7, 63.9)
Female	52 (89.7%)	16 (100.0%)	14 (82.4%)	22 (88.0%)
Years since PAH diagnosis	6.7 (2.3, 10.7)	7.5 (2.8, 12.0)	5.9 (1.2, 10.9)	6.8 (2.7, 10.0)
6MWD (m)	427.9 (362.1, 487.4) (N = 54)	413.8 (359.1, 461.0)	427.9 (351.1, 460.9) (N = 15)	457.2 (369.0, 526.7) (N = 23)
mPAP (mmHg)	40.3 (27.0, 49.3) (N = 35)	39.8 (31.8, 48.3) (N = 12)	29.2 (22.0, 37.0) (N = 10)	46.7 (40.3, 50.0) (N = 13)
PVR (WU)	5.1 (3.6, 6.3) (N = 31)	5.1 (3.9, 6.2) (N = 11)	3.6 (2.4, 3.7) (N = 9)	6.0 (5.6, 6.9) (N = 11)
DLCO (% predicted)	65.5 (53.0, 77.0) (N = 30)	53.0 (46.0, 72.0) (N = 10)	62.0 (53.0, 95.0) (N = 9)	77.0 (53.0, 94.0) (N = 11)
RVSP (mmHg)	50.0 (36.0, 61.0) (N = 31)	57.0 (31.0, 73.0) (N = 7)	37.0 (22.0, 49.0) (N = 11)	60.0 (47.0, 62.0) (N = 13)
ET-1 (pg/mL)	2.7 (2.3, 5.1) (N = 50)	3.1 (2.3, 3.9) (N = 14)	2.2 (1.8, 2.7) (N = 15)	4.4 (2.5, 6.0) (N = 21)
NT-proBNP (pg/mL)	289.0 (119.0, 592.0) (N = 53)	386.0 (119.0, 661.0) (N = 15)	264.0 (89.5, 541.0) (N = 16)	297.0 (125.0, 592.0) (N = 22)
RDW (%)	14.6 (13.9, 16.5) (N = 51)	15.1 (14.2, 16.4) (N = 15)	14.4 (13.5, 15.0) (N = 15)	15.4 (14.2, 16.8) (N = 21)
cGMP (pmoles/mL)	51.0 (36.0, 120.3) (N = 48)	41.3 (33.1, 109.8) (N = 13)	60.7 (33.3, 121.3) (N = 15)	51.0 (39.4, 115.7) (N = 20)
SNO-Hb (moles SNO/mole Hb)	0.0011 (0.0008, 0.0019) (N = 36)	0.0008 (0.0005, 0.0011) (N = 10)	0.0010 (0.0008, 0.0014) (N = 12)	0.0017 (0.0011, 0.0021) (N = 14)
cGMP to NTproBNP ratio (pmoles/pg)	0.25 (0.10, 0.63) (N = 43)	0.23 (0.10, 0.66) (N = 12)	0.29 (0.05, 0.69) (N = 14)	0.27 (0.11, 0.41) (N = 17)
ADMA (nM)	520.0 (446.0, 563.0) (N = 53)	550.5 (500.0, 556.0) (N = 14)	526.5 (442.5, 594.0) (N = 16)	515.0 (432.0, 565.0) (N = 23)
SDMA (nM)	413.0 (352.7, 493.5) (N = 50)	352.7 (327.0, 402.2) (N = 13)	453.6 (411.2, 521.0) (N = 14)	424.9 (353.6, 500.0) (N = 23)
NO2- (nM)	51.2 (34.1, 98.2) (N = 30)	72.6 (41.0, 163.4) (N = 10)	39.0 (26.5, 53.2) (N = 8)	46.9 (35.0, 93.0) (N = 12)

Continuous variables presented as median (25, 75); categorical variables presented as frequency (proportion)

ERA, endothelin receptor antagonist; PDE5i, phosphodiesterase 5 inhibitor; sGC, soluble guanylate cyclase stimulator; PAH, pulmonary arterial hypertension; 6MWD, six minute walk distance; m, meters; mPAP, mean pulmonary artery pressure; PVR, pulmonary vascular resistance; WU, woods units; RVSP, right ventricular systolic pressure

^a^2/17 (11.8%) patients on therapy with soluble guanylate cyclase stimulator, the remainder on PDE5i

^b^1/25 (4.0%) on therapy with soluble guanylate cyclase stimulator instead of PDE5i

In the sensitivity analysis, an additional 50 subjects were included in the cohort as follows: 17 patients with PAH and concurrent left heart disease (17/108, 15.7%), 20 patients with PAH and concurrent lung disease (20/108, 18.5%), and 13 patients with CTEPH (13/208, 12.0%). A similar proportion of patients with PAH and concomitant left heart disease were treated with an ERA (35%), PDE-5i (35%), or with the combination of an ERA and PDE5i (29%). A higher proportion of the patients with PAH and concomitant lung disease were treated with PDE5i (55%) as compared to treatment with ERA (10%) or the combination of an ERA and a PDE5i (29%). The majority of patients with CTEPH (62%) were treated with the combination of ERA and PDE-5i therapy in comparison to ERA monotherapy (15%) or PDE5i monotherapy (15%). Additional characteristics, along with baseline biomarker values, of these patients can be found in Additional file 1: Tables S1 and S2. Over the study period, a total of 56 PAH-related clinical events occurred in 36 subjects (Additional file 1: Table S3).

### Relationship between biomarkers and same-visit 6MWD

**Fig. 1:**
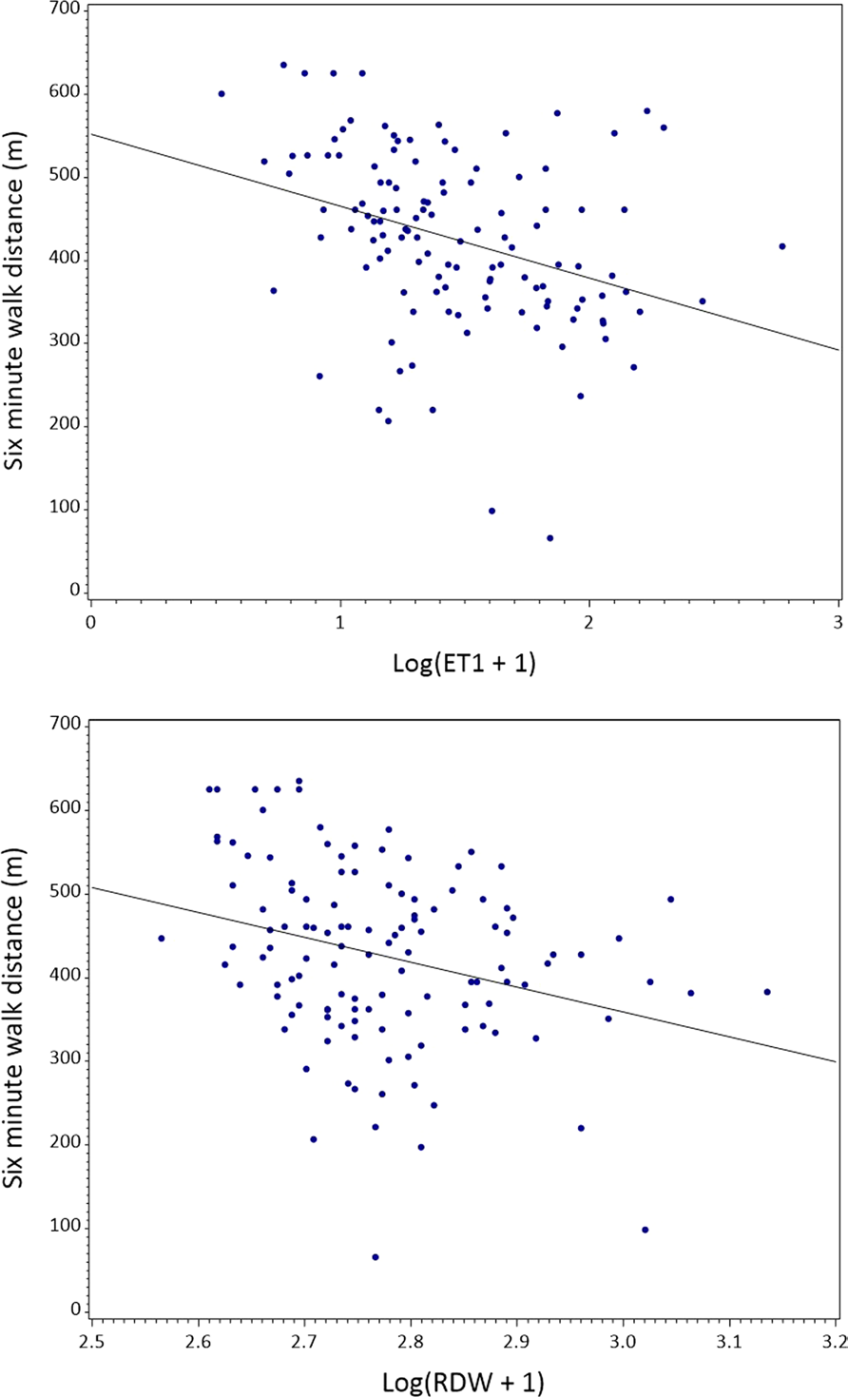
Univariable associations between 6MWD and ET-1 and RDW values at the same visit at study baseline

**Fig. 2:**
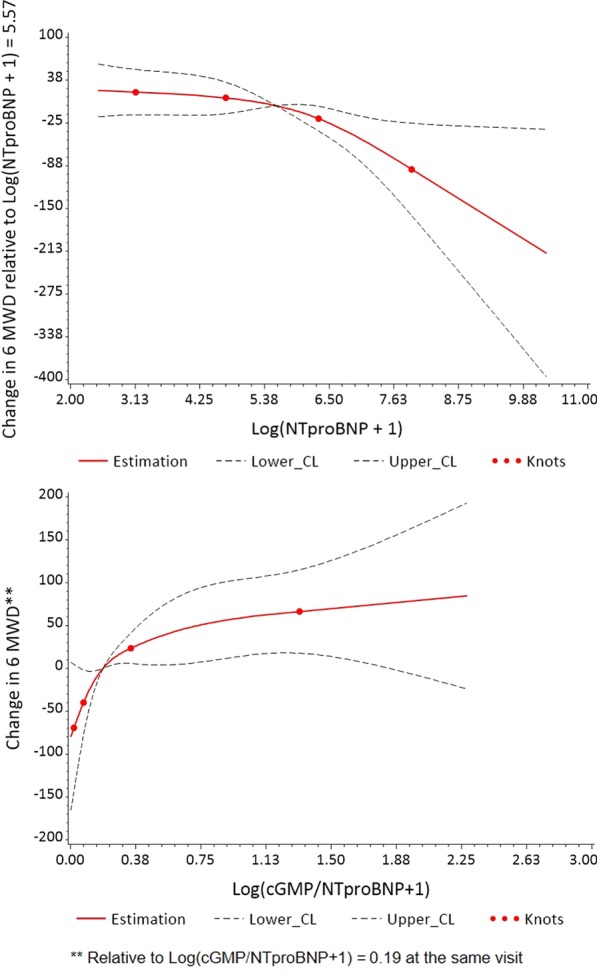
Restricted cubic splines plot showing the univariable association between 6MWD and NT-proBNP and cGMP/NT-proBNP ratio at the same visit at study baseline

In the overall cohort of 58 PAH patients, higher levels of ET-1 and RDW associated with a decreased 6MWD at the same visit (Table 2, Fig. 1). Elevations in NT-proBNP also associated with decreased 6MWD above a threshold of value of log(NT-proBNP + 1) > 5.5 (Fig. 2). In addition, the cGMP/NT-proBNP ratio associated with 6MWD below a threshold value of log(cGMP/NTproBNP + 1) = 0.3. Levels of SDMA had an inverse relationship with 6MWD above a threshold value of log(SDMA + 1) > 6, though this association did not meet criteria for significance (p = 0.07). In patients with PAH on ERA therapy, ADMA inversely associated with 6MWD (p = 0.01). Apart from ADMA, the relationship between all other biomarkers and 6MWD did not differ between treatment classes. There was no association between SNO-Hb, NO_2_^−^ and 6MWD.

**Table 2:** Relationship between 6MWD and Same-visit Biomarkers

Biomarker^a^	Estimate	Standard error	p-value
ET-1 (N = 55)	− 44.94	18.19	0.016
NTproBNP (N = 55)			
Log(NTproBNP + 1) ≤ 5.5, per 1 unit increase	− 4.67	7.37	0.53
Log(NTproBNP + 1) > 5.5, per 1 unit increase	− 31.25	8.71	0.0006
RDW (N = 55)	− 184.04	71.37	0.012
cGMP (N = 54)	1.78	10.57	0.87
SNO-Hb (N = 50)	1303.27	2822.37	0.64
cGMP/NTproBNP ratio (N = 53)			
Log(cGMP/NTproBNP + 1) ≤ 0.3, per 1 unit increase	180.89	82.37	0.032
Log(cGMP/NTproBNP + 1) > 0.3, per 1 unit increase	27.23	27.30	0.32
NO_2_^−^ (N = 49)	7.47	6.75	0.28
SDMA (N = 54)			
Log(SDMA + 1) ≤ 6, per 1 unit increase	5.82	46.70	0.90
Log(SDMA + 1) > 6, per 1 unit increase	− 46.96	25.25	0.067
ADMA (N = 54)^b^			
ADMA, PAH on treatment with ERA	− 0.22	0.087	0.012
ADMA, PAH on treatment with PDE5i	− 0.035	0.11	0.75
ADMA, PAH on treatment with ERA + PDE5i	0.098	0.088	0.27

^a^All biomarkers are transformed as log(biomarker + 1) with the exception of ADMA. All models were adjusted for age

^b^Interaction p = 0.038

Similar relationships between biomarkers and 6MWD were observed in a sensitivity analysis that included the larger cohort of 108 patients with pulmonary vascular disease on oral PAH therapy (Table 3). Specifically, higher levels of ET-1 and RDW associated with a decreased 6MWD, and similar relationships were seen with cGMP/NT-proBNP ratio and 6MWD. An analogous relationship was also observed with SDMA and 6MWD, with higher levels of SDMA associated with lower 6MWD after log(SDMA + 1) > 6 (p = 0.008). In patients with PAH and concomitant lung disease, ADMA inversely associated with 6MWD (p = 0.03). ADMA also inversely associated with 6MWD in patients with PAH on ERA therapy (p = 0.01). The association between NT-proBNP and 6MWD also varied by treatment class above a threshold value of log(NTproBNP + 1) > 5.5 (Table 3). Elevations in NT-proBNP associated with decreased 6MWD in patients with PAH on PDE-5i and ERA combination therapy, as well as patients with PAH with concomitant heart disease or CTEPH. There was no association between SNO-Hb, NO_2_^−^, or cGMP and 6MWD.

**Table 3:** Relationship between 6MWD and same-visit biomarkers in a larger cohort of subjects with pulmonary vascular disease

Biomarker^a^	Estimate	Standard error	p-value
ET-1 (N = 98)	− 33.51	13.82	0.017
NTproBNP (N = 99)			
Log(NTproBNP + 1) ≤ 5.5, per 1 unit increase	− 7.02	5.83	0.23
Log(NTproBNP + 1) > 5.5, per 1 unit increase^b^			
PAH on treatment with ERA	− 4.88	16.04	0.76
PAH on treatment with PDE5i	− 41.40	42.51	0.33
PAH on treatment with ERA + PDE5i	− 42.26	10.84	0.0002
PAH with concomitant left heart disease	− 43.89	18.84	0.021
PAH with concomitant lung disease	1.33	18.56	0.94
CTEPH	− 77.10	18.94	< .0001
RDW (N = 99)	− 179.30	54.84	0.001
cGMP (N = 96)	− 0.001	7.65	0.99
SNO-Hb (N = 92)	− 302.57	2498.01	0.90
cGMP/NTproBNP ratio (N = 94)			
Log(cGMP/NTproBNP + 1) ≤ 0.3, per 1 unit increase	205.01	59.11	0.0008
Log(cGMP/NTproBNP + 1) > 0.3, per 1 unit increase	8.48	18.33	0.64
NO2^−^ (N = 90)	1.85	5.49	0.74
SDMA (N = 97)			
Log(SDMA + 1) ≤ 6, per 1 unit increase	37.10	36.53	0.31
Log(SDMA + 1) > 6, per 1 unit increase	− 47.93	17.84	0.008
ADMA by therapy class and comorbidities (N = 98)^c^			
ADMA, PAH on treatment with ERA	− 0.23	0.086	0.01
ADMA, PAH on treatment with PDE5i	− 0.035	0.11	0.75
ADMA, PAH on treatment with ERA + PDE5i	0.098	0.086	0.26
ADMA, PAH with concomitant left heart disease	0.10	0.10	0.32
ADMA, PAH with concomitant lung disease	− 0.20	0.093	0.031
ADMA, CTEPH	− 0.20	0.16	0.21

^a^All biomarkers are transformed as log(biomarker + 1) with the exception of ADMA. All models were adjusted for age

^b^Interaction p = 0.028

^c^Interaction p = 0.032

To evaluate if models including multiple biomarkers would predict 6MWD better than any single biomarker alone, we created multivariable models to predict same-visit 6MWD using the baseline biomarkers in the larger cohort of patients with pulmonary vascular disease. This analysis yielded two possible multivariable models: (1) model including baseline cGMP/NT-proBNP ratio and SDMA (n = 67, R^2^ = 0.29) and (2) model including baseline NT-proBNP and SDMA (n = 76; R^2^ = 0.30, Additional file 1: Table S4). Both models had higher R^2^ values (explaining a greater proportion of the variance) than models with a single biomarker; however the varying number of patients used in two models prevents direct comparison in terms of fit.

### Relationship between biomarkers and future 6MWD

**Fig. 3:**
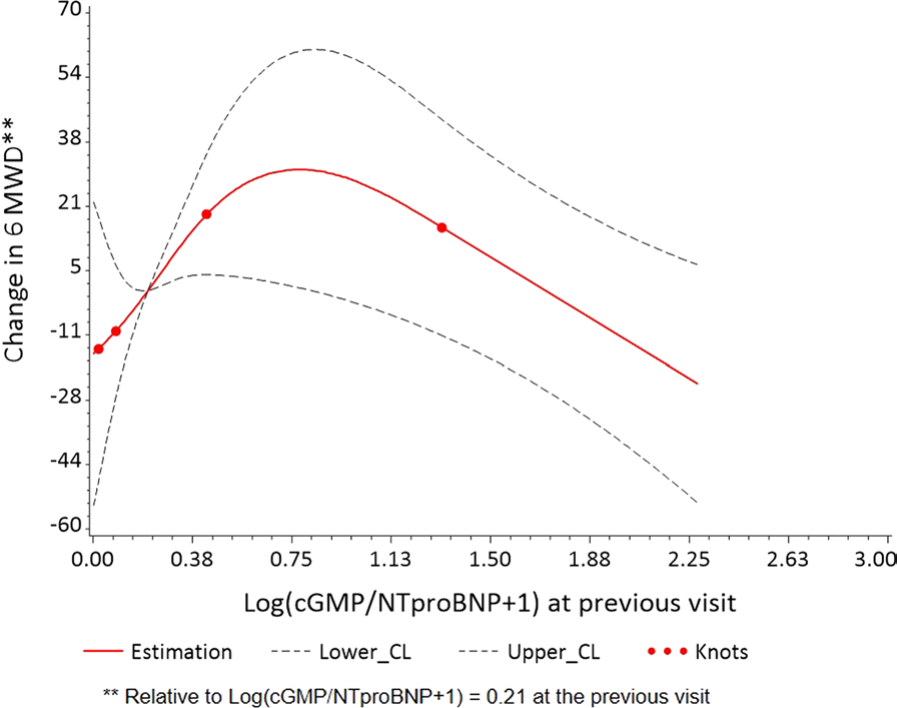
Univariable associations between 6MWD and cGMP/NTproBNP at the previous visit

ET-1 predicted a decreased 6MWD at the subsequent clinic visit (p = 0.02, Table 4). The cGMP/NT-proBNP ratio associated with an improved 6MWD below a threshold of log(cGMP/NT-proBNP + 1) = 0.75, though this relationship was also not significant (p = 0.08, Fig. 3). RDW, cGMP, ADMA, SDMA, and SNO-Hb did not associate with 6MWD at a future visit.

**Table 4:** Prediction of 6MWD Using Biomarker Value at Previous Visit in Patients with PAH

Biomarker^a^	Estimate	Standard error	Standardized model coefficients^b^	p-value
ET-1 (N = 48)	− 36.60	14.30	− 15.46	0.015
NT-proBNP (N = 49)	− 7.00	4.62	− 8.63	0.14
RDW (N = 49)	− 16.63	65.35	− 1.88	0.80
cGMP (N = 46)	− 8.51	12.22	− 5.05	0.49
SNO-Hb (N = 41)	− 5148.68	5365.76	− 3.69	0.35
cGMP/NTproBNP ratio (N = 44)				
Log(cGMP/NTproBNP + 1) ≤ 0.75^c^	59.91	33.47	25.21	0.083
Log(cGMP/NTproBNP + 1) > 0.75^c^	− 30.73	29.63	− 12.93	0.31
NO_2_^−^ (N = 41)	3.31	9.53	3.64	0.73
ADMA (N = 46)	− 0.0026	0.064	− 0.22	0.97
SDMA (N = 47)	− 23.97	17.87	− 7.62	0.19

^a^All biomarkers were transformed as log(biomarker + 1) besides ADMA

^b^Standarized model coefficients quantify change per 1 standard deviation increase in values of each biomarker

^c^Per 1 unit increase on log scale

In the sensitivity analysis that included the larger cohort of patients with pulmonary vascular disease, both ET-1 (p = 0.02) and RDW (p = 0.02) predicted decreased 6MWD at the subsequent visit (Additional file 1: Table S5, Figure S1). The cGMP/NT-proBNP ratio predicted an improved 6MWD at the next visit (p = 0.04) below a threshold of log(cGMP/NT-proBNP + 1) = 0.75.

## Discussion

Predicting clinical response to a given treatment is one of the most difficult challenges in PAH pharmacotherapy. A treatment-relevant biomarker could improve decision-making, and potentially, outcomes in PAH. Currently the only predictive biomarker used for PAH therapy selection is vasoreactivity, or acute vasodilation in the presence of inhaled NO during cardiac catheterization [16]. Our study is the first to assess the impact of mediators along current treatment pathways on clinical outcomes in a concerted, hypothesis-driven manner. We demonstrated that both ET-1 and the cGMP/NT-proBNP ratio associated with 6MWD at the same visit. Furthermore, ET-1 and the cGMP/NT-proBNP ratio predicted future 6MWD in a larger cohort of patients with pulmonary vascular disease that included patients with CTEPH, as well as PAH patients with concomitant heart or lung disease. Also in the larger cohort, ADMA associated with same visit 6MWD in patients with PAH on an ERA as well as patients with PAH and concomitant lung disease. In addition, SDMA associated with 6MWD at the same visit, regardless of medication class or concomitant heart or lung disease.

The value of using multiple biomarkers to improve risk assessment was recently demonstrated in a study by Rhodes and colleagues, who identified a nine-protein panel that predicted survival in PAH patients [17]. The functions of the proteins identified by Rhodes et al*.* related to myocardial stress, inflammation, and pulmonary vascular cell dysfunction [17]. Given the complex interactions among mediators within the biological pathways that influence pulmonary vascular remodeling and vasoreactivity [13], the parallel study of putative mediators as biomarkers may have intrinsic advantages and predictive power as compared to their study individually. Illustrating the web of related mediators is the interaction between cGMP and NT-proBNP. While cytosolic cGMP is produced in response to nitric oxide (NO), membrane-derived cGMP is produced in response to members of the natriuretic peptide (including ANP and BNP) family, and thus cGMP elevations may parallel those in BNP rather than in response to NO [11, 18, 19]. This could explain the varying and unexpected relationships in treatment-induced changes in cGMP levels to clinical endpoints seen in previous studies [18, 20]. Therefore we were particularly interested in the cGMP/NT-proBNP ratio, as a biomarker of treatment response. Our finding that cGMP/NTproBNP ratio predicted 6MWD at a subsequent clinic visit is novel, has potential theranostic value, and will need to be validated in future studies.

ET-1 is a potent vasoconstrictor that also stimulates proliferation of pulmonary artery smooth muscle cells. Elevated plasma ET-1 levels previously predicted clinical worsening in idiopathic PAH patients on bosentan therapy [22], but it is unknown whether ET-1 levels predict therapeutic response in a broader PAH cohort. Interestingly, plasma levels of ET-1 and BNP have been significantly correlated in patients with PAH, generating the hypothesis that increased levels of ET-1 may result from systemic neuro-hormonal activation [21]. ERA therapy is known to acutely increase ET-1 levels in patients with PAH [23], reflecting an incompletely characterized neuro-hormonal or other feedback regulation loop, although the elevations in ET-1 levels may fade with chronic ERA therapy [21]. In our cohort, ET-1 levels were lowest in patients with PAH on PDE-5i, and highest in patients on the combination of ERA and PDE-5i therapies. Our finding that elevations in ET-1 associated with a lower same-visit 6MWD is consistent with prior work by Rubens and colleagues [24]. A novel finding in the present study is that elevations in ET-1 also predicted a decreased 6MWD at the subsequent clinic visit. Interestingly, the correlation between ET-1 and same-visit or future 6MWD was similar among patients on ERA monotherapy, patients on PDE-5i monotherapy, and patients on combination PDE-5i and ERA therapy, though we note that the power was limited to detect differences according to the oral therapy classes.

To our knowledge, this is the first prospective cohort study to investigate associations between SDMA and clinical outcomes in patients with PAH. The arginine derivatives SDMA and ADMA effectively interfere with NO synthesis by competing with L-arginine for the active site of NO synthase, and have been proposed as biomarkers in PAH and other cardiovascular diseases [25, 26]. Elevated SDMA has previously associated with renal dysfunction [27] and increased mortality in patients with coronary artery disease [28], atrial fibrillation [26], and stroke [29]. In the current study, we demonstrated that increased levels of SDMA associated with worsening 6MWD at the same visit after a threshold of log(SDMA + 1) = 6 in the larger cohort of patients with pulmonary vascular disease. A similar inflection point with SDMA and clinical outcomes has also been seen in previous studies in cardiovascular disease [26, 28]. This threshold effect might result when a critical SDMA level capable of competing with l-arginine is reached.

A more potent inhibitor of NO synthesis than SDMA [30], ADMA has been shown to associate with hemodynamic indices and survival in prior studies of PAH patients [25, 31]. In the current study, we found that that high ADMA levels associated with lower 6MWD in PAH patients on ERAs but not in PAH patients on either PDE-5i or on PDE-5i/ ERA combination therapy. One possible explanation is that the presence of PDE-5i may increase cGMP levels to help compensate for decreased NO signaling in patients with high levels of ADMA. High ADMA levels in patients on ERA therapy might therefore help identify those patients most likely to benefit from initiation of PDE-5i (as opposed to those patients in whom combination therapy adds only exposure risk and no benefit). In our study high ADMA levels also associated with lower 6MWD in PAH patients with concomitant lung disease. This could be because hypoxia causes a decrease in expression of dimethyl-arginine dimethylaminohydrolase (DDAH), the enzyme that metabolizes ADMA [32]. As PAH patients with concurrent lung disease are likely to have hypoxemia, ADMA levels may be higher in PAH patients with lung disease compared to other PAH patients. As a result, ADMA may play a bigger role in the pathogenesis of PAH in patients with lung disease.

Other mediators evaluated along the NO pathway, including SNO-Hb and NO_2_^−^, did not associate with 6MWD in this study. This may be because NO_2_^−^ is also determined by other factors including diet and glomerular filtration, contributing to the variable levels of NO_2_^−^ in patients with PAH in other studies [33–35].

Important strengths of our study include the concerted study of multiple biomarkers, over time, in patients with PAH. In many previous studies, potentially relevant biomarkers have been examined or at least reported only individually [13, 36], which limits the ability to account for the cross-talk in these biological systems. Another strength of this study is that we successfully employed a protocol ensuring that potentially labile analytes such as NO_2_^−^, SNO-Hb, cGMP, and ET-1, were protected from light; preserved in low-nitrite, opaque (foil-covered) tubes on ice; and immediately transported to the analytical lab and processed within 120 min. Finally, the inclusion of patients with PAH with concomitant heart and lung disease reflects contemporary, real-world practice of PAH treatment.

This study has several important limitations. We studied only prevalent PAH patients, precluding the ability to determine changes in biomarkers before and after oral therapy. As patients are increasingly being started on upfront ERA-PDE5i combination therapy [3], prevalent PAH patients are an important population to study in order to understand how to titrate therapies. Patients on parenteral therapy tend to have more advanced disease, and because we excluded such patients, our cohort was a relatively healthy one with slower progression of disease. We therefore do not know the role these biomarkers might play in predicting such rapid progression. By the same token, rapid deterioration in a PAH patient typically leads to intensification of therapy that often includes addition of intravenous or other parenteral prostanoid therapy. Therefore biomarker data relevant to the choice of oral therapy class in this setting is unlikely to provide additional actionable information.

Another limitation in this study is the heterogeneity of the cohort, and as such the sample size in each therapy class subgroup may have lacked power to detect an interaction. Notably, given the diagnostic algorithm used to identify PAH patients with concomitant left heart disease or lung disease and to distinguish them from WHO Groups 2 and 3 respectively, the treating clinicians viewed this subset of patients as having PAH that was not attributable to (or was “out of proportion to”) their heart or lung disease, leading to the initiation of oral therapy and therefore eligibility for enrollment in this study. In our analysis of patients with heart disease, lung disease, and CTEPH treated with oral therapy for concomitant PAH, we performed an interaction analysis, which allowed for the relationship between biomarker value and 6MWD to differ among the groups. Our study may have lacked the power needed to detect differences in biomarkers, and similar analyses in larger cohorts of patients may better clarify underlying biological and treatment-dependent outcome differences amongst different phenotypes. We also performed an analysis of PAH patients without concomitant heart or lung disease because of this important consideration. But even “pure” PAH is a heterogeneous disease with regards to age, gender, disease severity, etiology, and genetic background, and the pathobiologic mechanisms may differ among the individuals in our PAH cohort and in others. This emphasizes the need for continued study and treatment-relevant phenotyping of all patients with pulmonary vascular disease.

As this is an exploratory study, no adjustment was made for the multiple testing performed and the associations identified must therefore be interpreted cautiously and require validation in a separate cohort. Also, the use of a single variable (6MWD) may limit the ability to determine associations between biomarker and disease. However, other measures of disease severity, including echocardiography or pulmonary hemodynamic measurements were not performed as frequently in this cohort of patients on oral PAH therapies. Finally, routinely performed lab values such as NT-proBNP were available to treating physicians and were likely considered in the clinical management of patients. This may weaken any natural association between the biomarker and clinical outcomes.

## Conclusions

In summary, we identified several biomarkers along current treatment pathways that correlate with clinical outcomes in patients with PAH. Specifically, ET-1, cGMP/NT-proBNP ratio, and SDMA all associate with 6MWD. Furthermore, the relationship between ADMA and 6MWD differs among oral therapy classes. Given the complexity of the interactions among circulating mediators that can serve as treatment-related biomarkers, studying them in concert and over time has clear advantages. These findings offer a first step toward a clinically useful panel of biomarkers that could help clinicians select therapy and assess response. In future studies, it would be rational to test whether outcomes may be improved using a strategy of assignment of patients with moderately severe PAH to a given treatment class based on a biomarker or panel of biomarkers.

## Supplementary information

**Additional file 1.** Additional tables and figures.

## Data Availability

The datasets used and/or analysed during the current study are available from the corresponding author on reasonable request.

## Abbreviations

6MWD: Six minute walk distance
ADMA: Asymmetric dimethylarginine
BNP: Brain natriuretic peptide
cGMP: Cyclic guanosine monophosphate
CTEPH: Chronic thromboembolic pulmonary hypertension
EDTA: Ethylenediamine tetraacetic acid
ERA: Endothelin receptor antagonist
ET: Endothelin
NO: Nitric oxide
NO_2_^−^: Nitrite
NT-proBNP: N-terminal fragment of pro-brain natriuretic peptide
PAH: Pulmonary arterial hypertension
PH: Pulmonary hypertension
PDE5i: Phosphodiesterase 5 inhibitor
RBC: Red blood cell
RDW: Red blood cell distribution width
SDMA: Symmetric dimethylarginine
sGC: Soluble guanylate cyclase
SNO-Hb: *S*-Nitrosohemoglobin
