# Supplementary material for: Treatment-related biomarkers in pulmonary hypertension patients on oral therapies
Source: Respir Res. 2020 Nov 19;21:304. doi: 10.1186/s12931-020-01566-y (PMC7678114; doi:10.1186/s12931-020-01566-y)
Supplement: Supplementary file 1 — Additional file 1. Additional tables and figures. [file 12931_2020_1566_MOESM1_ESM.docx]

| Additional file 1: Table S1: Baseline Patient Characteristics in the Overall Sensitivity Cohort,  and as Stratified by Therapy Class and Additional PH Risk Factors | | | | | | | |
| --- | --- | --- | --- | --- | --- | --- | --- |
|  | **Overall**  **(N = 108)** | **PAH on therapy with ERA**  **(N = 16)** | **PAH on therapy with PDE5i**  **(N = 17)** | **PAH on therapy with combination ERA and PDE5i**  **(N = 25)** | **PAH with concomitant left heart disease**  **(N =17)** | **PAH with concomitant lung disease**  **(N = 20)** | **CTEPH**  **(N = 13)** |
| Age (yrs) | 60.4±14.4 | 59.9±14.1 | 65.6±13.9 | 49.9±15.5 | 66.1±5.8 | 61.2±11.8 | 65.4±15.9 |
| Years with  PH | 6.2±5.4 | 7.7± 5.5 | 6.8±6.1 | 7.8±7.2 | 5.1±3.8 | 4.3±2.8 | 5.0±3.9 |
| Female | 91 (84%) | 16 (100%) | 14 (82%) | 22 (88%) | 15 (88%) | 15 (75%) | 9 (69%) |
| 6MWD (m) | 386 ± 113  N = 92 | 415 ± 67 | 414 ± 96  N=15 | 444 ± 96  N=23 | 290 ± 108  N=9 | 325 ± 108  N=17 | 357 ± 143  N=12 |
| mPAP*  (mmHg) | 39.4± 12.5  N = 57 | 39.5± 12.0  N = 12 | 30.2± 12.8  N = 10 | 43.4± 11.2  N = 13 | 41.5± 7.9  N = 8 | 39.0± 14.4  N = 9 | 44.9± 14.4  N = 5 |
| PVR (WU)* | 5.4±3.0  N = 53 | 4.9±1.7  N = 11 | 3.3±1.7  N = 9 | 6.9 ±3.4  N = 11 | 4.9 ±2.8  N = 8 | 6.5±4.0  N = 9 | 6.0 ±2.7  N = 5 |
| DLCO* (% predicted) | 60±26  N = 53 | 56±17  N = 10 | 72±27  N = 9 | 72±29  N = 11 | 59±22  N = 4 | 40±18  N = 12 | 66±27  N = 7 |
| RVSP*  (mmHg) | 51±18  N = 58 | 56±20  N =7 | 38±15  N = 11 | 55±13  N = 13 | 50±22  N = 8 | 52±23  N = 11 | 55±15  N = 8 |
| PH Therapy  ERA  PDE5i  ERA+PDE5i  sGC | 26 (24%)  34 (32%)  44 (41%)  4 (4%) | 16 (100%)  0 (0%)  0 (0%)  0 (0%) | 0 (0%)  15 (88%)  0 (0%)  2 (12%) | 0 (0%)  0 (0%)  24 (96%)  1 (4%) | 6 (35%)  6 (35%)  5 (29%)  0 (0%) | 2 (10%)  11 (55%)  7 (35%)  0 (0%) | 2 (15%)  2 (15%)  8 (62%)  1 (8%) |
| WHO = World Health Organization; CpC = combined post and pre-capillary; PH = pulmonary hypertension; 6MWD = six minute walk distance; m = meters; mPAP = mean pulmonary artery pressure; PVR = pulmonary vascular resistance; WU = woods units; RVSP = right ventricular systolic pressure  Continuous variables presented as mean ±SD; categorical variables presented as frequency (proportion)  * Data presented from the right heart catheterization, echocardiogram, or pulmonary function test closest to enrollment are presented. | | | | | | | |

| Additional file 1: Table S2: Baseline Biomarker Values in the Entire Cohort,  and as Stratified by Therapy Class and Additional PH Risk Factors | | | | | | | |
| --- | --- | --- | --- | --- | --- | --- | --- |
| Baseline  biomarker | Group 1,  ERA | Group 1,  PDE5i | Group 1,  ERA+PDE5i | PAH with concomitant  left heart disease | PAH with concomitant lung disease | CTEPH | Overall |
| ET-1 (pg/mL) | 3.1 (2.3, 3.9)  N = 14 | 2.2 (1.8, 2.7)  N = 15 | 4.4 (2.5, 6.0)  N = 21 | 3.0 (2.0, 5.9)  N = 15 | 2.8 (1.5, 4.0)  N = 19 | 3.0 (2.0, 5.4)  N = 11 | 2.8 (2.2, 5.1)  N = 95 |
| NTproBNP  (pg/mL) | 386.0 (119.0, 661.0)  N = 15 | 264.0 (89.5,  541.0)  N = 16 | 297.0 (125.0,  592.0)  N = 22 | 699.0 (261.0, 1301.0)  N = 17 | 244.5 (100.0,  462.5)  N = 20 | 461.0 (192.0, 837.0)  N = 13 | 302.0 (122.0, 747.0)  N = 103 |
| RDW (%) | 15.1 (14.2,  16.4)  N = 15 | 14.4 (13.5,  15.0)  N = 15 | 15.4 (14.2,  16.8)  N = 21 | 15.4 (14.4,  16.3)  N = 17 | 14.7 (14.1,  15.5)  N = 20 | 14.3 (13.7,  15.8)  N = 13 | 14.7 (14.0,  16.1)  N = 101 |
| cGMP  (pmoles/mL) | 41.3 (33.1, 109.8)  N = 13 | 60.7 (33.3, 121.3)  N = 15 | 51.0 (39.4,  115.7)  N = 20 | 48.7 (36.1,  85.0)  N = 15 | 44.5 (39.5,  101.2)  N = 19 | 59.5 (36.2,  133.8)  N = 11 | 49.2 (38.3, 107.9)  N = 93 |
| SNO-Hb (moles SNO/mole Hb) | 0.0008  (0.0005,  0.0011)  N = 10 | 0.0010  (0.0008,  0.0014)  N = 12 | 0.0017  (0.0011,  0.0021)  N = 14 | 0.0006  (0.0004,  0.0007)  N = 14 | 0.0006  (0.0004,  0.0011)  N = 13 | 0.0008  (0.0008,  0.0023)  N = 11 | 0.0009  (0.0006,  0.0015)  N = 74 |
| cGMP to NTproBNP ratio (pmoles/pg) | 0.23  (0.10, 0.66)  N = 12 | 0.29  (0.05, 0.69)  N = 14 | 0.27  (0.11, 0.41)  N = 17 | 0.07  (0.02, 0.49)  N = 15 | 0.21  (0.10, 0.46)  N = 19 | 0.10  (0.06, 0.74)  N = 11 | 0.20  (0.07, 0.49)  N = 88 |
| ADMA (nM) | 550.5  (500.0, 556.0)  N = 14 | 526.5  (442.5, 594.0)  16 | 515.0  (432.0, 565.0)  N = 23 | 549.5  (473.0, 643.0)  N = 16 | 523.0  (467.0, 614.0)  N = 17 | 551.0  (458.0, 607.0)  N = 11 | 523.0  (457.0, 576.0)  N = 97 |
| SDMA (nM) | 352.7 (327.0, 402.2)  N = 13 | 453.6 (411.2, 521.0)  N = 14 | 424.9 (353.6, 500.0)  N = 23 | 502.5 (423.2, 785.5)  N = 16 | 543.0 (407.6, 621.0)  N = 17 | 534.5 (377.0, 737.0)  N = 10 | 435.0 (377.0, 561.0)  N = 93 |
| NO_2_^-^ (nM) | 72.6  (41.0, 163.4)  N = 10 | 39.0  (26.5, 53.2)  N = 8 | 46.9  (35.0, 93.0)  N = 12 | 62.7  (48.1, 84.1)  N = 15 | 41.2  (35.3, 85.4)  N = 13 | 78.3  (44.3, 116.6)  N = 10 | 56.2  (36.2, 94.9)  N = 68 |
| ET-1 = endothelin-1; NTproBNP = N-terminal fragment of brain natriuretic peptide; RDW = red cell distribution width; cGMP = cyclic guanosine monophosphate; SNO-Hb = S-nitrosohemoglobin; ADMA= asymmetric dimethylarginine; SDMA = symmetric dimethylarginine; NO_2_^-^ = nitrite  Continuous variables presented as median (IQR) | | | | | | | |

| Additional file 1: Table S3: PH-Related Clinical Events* | |
| --- | --- |
| Event Type | **Number of Events (%)** |
| Death | 11 (20%) |
| PH – related hospital stay | 13 (23%) |
| Lung Transplant | 2 (4%) |
| Initiation of SQ or IV Prostacyclin | 6 (11%) |
| Initiation of inhaled Prostacyclin | 11 (20%) |
| Addition of oral therapy due to clinical worsening  PDE5i  ERA  Riociguat | 13 (23%)  6 (11%)  5 (9%)  2 (4%) |
| ** A total of 56 clinical events were experienced by 36 patients.* | |

| Additional file 1: Table S4: Multivariable Models evaluating Relationship between 6MWD and Biomarkers at Study Baseline | | | | |
| --- | --- | --- | --- | --- |
| *Multivariable Models* (Number of patients) | Estimate | Standard Error | P-value | R^2 $^ |
| Model 1 (N = 67)  Log(cGMP/NTproBNP + 1)  Log(SDMA + 1) >6, per 1 unit increase | 89.75  -115.68 | 27.36  40.36 | 0.002  0.006 | 0.29 |
| Model 2 (N = 76)  Log(NTproBNP + 1)  Log(SDMA + 1) >6, per 1 unit increase | -27.82  -144.78 | 9.70  40.25 | 0.005  0.001 | 0.30 |
| ET-1 = endothelin-1; NTproBNP = N-terminal fragment of brain natriuretic peptide; RDW = red cell distribution width; cGMP = cyclic guanosine monophosphate; SNO-Hb = S-nitrosohemoglobin; ADMA= asymmetric dimethylarginine; SDMA = symmetric dimethylarginine; NO_2_^-^ = nitrite  *All biomarker values are transformed as log(biomarker +1), apart from ADMA  ^$^ R^2^ values are shown for single biomarkers that are also in the multivariable model | | | | |

| Additional file 1: Table S5: Prediction of 6MWD Using Biomarker Value at Previous Visit in larger cohort of pulmonary vascular disease subjects | | | | |
| --- | --- | --- | --- | --- |
| Biomarker* | **Estimate** | **Standard Error** | **Standardized model coefficients^$^** | **P-value** |
| ET -1 (N = 79) | -30.04 | 12.14 | -13.90 | 0.02 |
| NT-proBNP (N = 82) | -7.08 | 3.85 | -9.14 | 0.07 |
| RDW (N = 82) | -128.88 | 52.73 | -12.76 | 0.02 |
| cGMP (N = 77) | 3.29 | 9.11 | 1.85 | 0.72 |
| SNO-Hb (N = 70) | -976.15 | 4446.26 | -0.78 | 0.83 |
| cGMP/NTproBNP ratio  Log (cGMP/NTproBNP + 1) ≤ 0.75 ^ϕ^  Log (cGMP/NTproBNP + 1) >0.75 ^ϕ^ | 50.38 -12.25 | 23.91 23.57 | 20.18  -4.91 | 0.04 0.61 |
| NO_2_^-^ (N = 72) | -1.46 | 7.99 | -1.26 | 0.86 |
| ADMA (N = 78) | 0.002 | 0.05 | 0.17 | 0.97 |
| SDMA (N= 79) | 3.56 | 13.24 | 1.41 | 0.79 |
| * All biomarkers were transformed as log(biomarker+1) besides ADMA. All models were adjusted for age  ^$^ Standarized model coefficients quantify change per 1 standard deviation increase in values of each biomarker  ^ϕ^ Per 1 unit increase on log scale | | | | |

Additional file 1: **Figures**

**Figure S1: Association between 6MWD and Selected Biomarkers at the Previous Visit in larger cohort of pulmonary vascular disease subjects**

p=0.04

p=0.02

p=0.04
